# Muscle and not neuronal biomarkers correlate with severity in spinal and bulbar muscular atrophy

**DOI:** 10.1212/WNL.0000000000007097

**Published:** 2019-03-12

**Authors:** Vittoria Lombardi, Giorgia Querin, Oliver J. Ziff, Luca Zampedri, Ilaria Martinelli, Carolin Heller, Martha Foiani, Cinzia Bertolin, Ching-Hua Lu, Bilal Malik, Kezia Allen, Carlo Rinaldi, Henrik Zetterberg, Amanda Heslegrave, Linda Greensmith, Michael Hanna, Gianni Soraru, Andrea Malaspina, Pietro Fratta

**Affiliations:** From the Institute of Neurology (V.L., O.J.Z., L.Z., C.H., M.F., C.-H.L., B.M., H.Z., A.H., L.G., M.H., P.F.), University College London Institute of Neurology, Queen Square, London; Blizard Institute (V.L., A.M.), Queen Mary, University of London, UK; Department of Neurosciences (G.Q., I.M., C.B., G.S.), University of Padova, Italy; Department of Neurology (C.-H.L.), China Medical University Hospital, Taiwan; Basildon Hospital (K.A.), UK; Department of Physiology, Anatomy and Genetics (C.R.), University of Oxford; UK Dementia Research Institute at UCL (H.Z., A.H.), London, UK; Clinical Neurochemistry Laboratory (H.Z.), Sahlgrenska University Hospital; and the Department of Psychiatry and Neurochemistry (H.Z.), Institute of Neuroscience and Physiology, the Sahlgrenska Academy at the University of Gothenburg, Mölndal, Sweden.

## Abstract

**Objective:**

To determine whether blood biomarkers of neuronal damage (neurofilament light chain [NfL]), muscle damage (creatine kinase [CK]), and muscle mass (creatinine) are altered in spinal and bulbar muscular atrophy (SBMA) and can be used as biomarkers for disease severity.

**Methods:**

In this multicenter longitudinal prospective study, plasma and serum were collected from 2 cohorts of patients with SBMA in London, United Kingdom (n = 50), and Padova, Italy (n = 43), along with disease (amyotrophic lateral sclerosis [ALS]) and healthy controls, and levels of plasma and serum NfL, CK, and creatinine were measured. Disease severity was assessed by the SBMA Functional Rating Scale and the Adult Myopathy Assessment Tool at baseline and 12 and 24 months.

**Results:**

Blood NfL concentrations were increased in ALS samples, but were unchanged in both SBMA cohorts, were stable after 12 and 24 months, and were not correlated with clinical severity. Normal NfL levels were also found in a well-established mouse model of SBMA. Conversely, CK concentrations were significantly raised in SBMA compared with ALS samples, and were not correlated to the clinical measures. Creatinine concentrations were significantly reduced in SBMA, and strongly and significantly correlated with disease severity.

**Conclusions:**

While muscle damage and muscle mass biomarkers are abnormal in SBMA, axonal damage markers are unchanged, highlighting the relevant primary role of skeletal muscle in disease pathogenesis. Creatinine, but not CK, correlated with disease severity, confirming its role as a valuable biomarker in SBMA.

Spinal and bulbar muscular atrophy (SBMA), also known as Kennedy disease (KD), is a disabling adult onset neuromuscular disorder that affects men and is primarily characterized by slowly progressive weakness and wasting of bulbar and limb muscles.^1,2^ SBMA is caused by the expansion of a CAG repeat in the androgen receptor (AR) gene and is characterized by muscle denervation and loss of lower motor neurons in the spinal cord and the brainstem. Signs of primary skeletal muscle damage, such as muscle fiber splitting, fiber degeneration, and centralized nuclei, are also present in SBMA, and recent work showing that silencing of the mutation in muscle is able to rescue the disease phenotype in disease models has further highlighted the relevance of a primary myopathic component in this disorder.^3–9^

No therapy is currently available for SBMA and an important limitation to trials for promising therapeutic strategies has been the lack of effective outcome measures. Biomarkers to measure disease progression and therapeutic responses are therefore strongly needed.

Neurofilament light chain (NfL) has been found to be increased in serum and plasma of numerous neurologic conditions, including amyotrophic lateral sclerosis (ALS) and inherited peripheral neuropathies, and to be a promising tool to monitor disease progression.^10,11^ We here use the currently most sensitive methodology to measure NfL in patients with SBMA and in a rodent model of disease,^12^ and compare this marker of neuronal damage with measures of muscle damage and loss.

## Methods

### Standard protocol approvals, registrations, and patient consents

Plasma and serum were prospectively collected with informed consent from 2 cohorts of patients with a genetically confirmed diagnosis of SBMA attending the KD clinic at the National Hospital for Neurology in London, United Kingdom (n = 50), and at the University Hospital in Padova, Italy (n = 43). Samples from patients with ALS (n = 53) and healthy relatives of patients (n = 73) were collected in neuromuscular clinics in the same centers.

Approvals were obtained from the East London and the City Research Ethics Committee (09/H0703/27). Written informed consent was obtained from all participants in the study.

### Patient evaluation

All patients were recruited in neuromuscular clinics at the National Hospital for Neurology in London, United Kingdom, and at the University Hospital in Padova, Italy. All participants with SBMA carried a pathogenic expansion of the CAG trinucleotide repeat (>38 repeats) in the *AR* gene. ALS cases all were recruited according to El Escorial probable and definite ALS criteria. Controls were recruited in both centers in order to reduce sources of bias. Healthy controls were excluded if they had coexistent neurologic disease as determined by a symptom and medical history–based questionnaire.

SBMA and ALS disease severity and progression were evaluated using functional rating scale scores (Spinal and Bulbar Muscular Atrophy Functional Rating Scale [SBMAFRS], Amyotrophic Lateral Sclerosis Functional Rating Scale [ALSFRS]), and patients with SBMA were further evaluated with the Adult Myopathy Assessment Tool (AMAT).^13–15^

Progression rate in patients with ALS was calculated as progression rate to last visit (PRL). This was the ALSFRS–revised (range 1–48, with lower scores corresponding to higher level of neurologic impairment) approximated to 48 at onset minus the score at last visit divided per disease duration expressed in month (ALS-Fast = PRL >1; ALS-Slow = PRL <0.5).

### Sample collection and processing

Samples were processed, stored, and analyzed as previously described.^10,11^ Blood samples from all participants were collected into EDTA-containing tubes and centrifuged at 20°C at 3,500 rpm for 10 minutes within 1 hour. Repeat samples were taken after 1 and 2 years, when available. Plasma and serum were aliquoted and stored at −80°C.

### Mouse samples

All experimental procedures were carried out under license from the UK Home Office (Scientific Procedures Act 1986), and following approval by the Ethical Review Panel of UCL Institute of Neurology. Yeast artificial chromosome (YAC) AR100 mice, which express at endogenous levels the human androgen receptor with an expanded CAG repeat,^12^ were bred and maintained at UCL Institute of Neurology Biological Services. Heterozygote males carrying the androgen receptor with 100 polyglutamine repeats (pathogenic AR100 mice) were mated with wild-type C57BL/6J females. Only male mice were used in this study and male age-matched wild-type littermates were used as controls. Mouse plasma was obtained from male AR100 mice and controls at 18 months of age, as previously described.^12,16^

### NfL, creatine kinase (CK), and creatinine measurements

The quantitative determination of NfL in human plasma (UK cohort) and serum (Italian cohort) was undertaken by single molecule array (Simoa) technology using a digital immunoassay Simoa HD-1 Analyzer (Quanterix; Lexington, MA) using a commercially available NfL kit from the same vendor. Plasma samples from KD, ALS-Fast, ALS-Slow, and healthy patients, along with KD/WT mice, were equally distributed on each plate and measured in duplicate. Each plate contained a target-specific calibrator (500–0.686 pg/mL) and 2 quality controls (10 and 200 pg/mL).

In the first step, anti-NfL-antibody–coated paramagnetic capture beads, sample, and biotinylated detector antibody are combined. NfL molecules present in the sample are captured by the anti-NfL-antibody–coated capture beads and labeled with biotinylated detector antibodies. After washing, a conjugate of streptavidin-β-galactosidase (SBG) is mixed with the capture beads. SBG binds to the biotinylated detector antibodies, resulting in enzyme labeling of captured NfL. CK and creatinine were measured as part of routine clinical testing.

### Statistical analysis

Statistical analysis was performed using GraphPad Prism 5.0 (GraphPad; La Jolla, CA). Patient NfL, CK, and creatinine concentrations were compared using analysis of variance and Bonferroni correction for multiple testing. Mouse NfL levels were compared using Mann-Whitney test. Correlations of CK and creatinine levels with SBMAFRS and AMAT scores were assessed using Spearman correlation coefficients. We included all available samples for this study, given the rarity of the disease and the unknown variability and changes of NfL in SBMA prior to this work. Missing data were omitted from analysis—all numbers for each analysis are reported in the table.

**Table:**
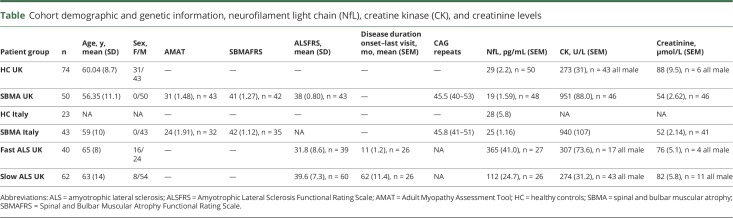
Cohort demographic and genetic information, neurofilament light chain (NfL), creatine kinase (CK), and creatinine levels

### Data availability

Anonymized data not published within this article will be made available by request from any qualified investigator.

## Results

### Cohorts

The study included 93 patients with SBMA, 53 patients with ALS, and 73 healthy controls. Participant characteristics are summarized in the table.

### NfL levels are unchanged in 2 cohorts of patients with SBMA

**Figure 1:**
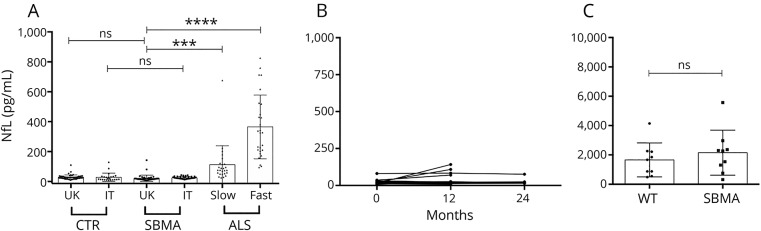
Neurofilament light chain (NfL) levels are unchanged in spinal and bulbar muscular atrophy (SBMA) (A) NfL concentrations (pg/mL) from the UK cohort (plasma, UK control [CTR], UK SBMA, slow amyotrophic lateral sclerosis [ALS], and fast ALS) and the Italian cohort (IT CTR and IT SBMA). Assays were conducted together, but statistical analysis (analysis of variance, Bonferroni multiple comparison correction) is represented only between samples from the same cohort. (B) NfL concentrations in the longitudinal study in the UK cohort. Twelve-month (n = 28) and 24-month (n = 8) timepoints represent 12 ± 2 and 24 ± 2 months. (C) NfL levels from AR100 (SBMA) and littermate control (WT) mice are shown (Mann-Whitney test). Columns indicate mean, error bars indicate SD; ***p < 0.001; ****p < 0.0001; ns *p* > 0.05.

We measured the plasma levels of NfL, using the highly sensitive Simoa assay, in samples from the UK SBMA cohort, and compared them with healthy controls and with slow and fast progressor ALS cases, as positive controls. While fast and slow ALS progressors showed the previously described^11^ increase in NfL levels (*p* < 0.0001 and *p* = 0.0008, respectively), we detected no significant increase in the SBMA cases (*p* = 0.99). This finding was replicated in the Italian SBMA cohort (*p* > 0.99) (figure 1A). We further examined whether levels changed longitudinally at 12 (n = 28) and 24 (n = 8) months, and showed NfL levels to be stable across this time, with the exception of 3/28 cases where levels increased to >58 pg/mL, the 95th percentile of controls (figure 1B).

### NfL levels are unchanged in a mouse model of disease

We assessed NfL levels in a well-established SBMA mouse model: AR100 mice. These mice express a 100-CAG expansion in the human AR and develop a late-onset progressive neuromuscular phenotype characterized by muscle atrophy and motor neuron degeneration, with no motor neuron loss at 6 months, and a 40% loss of motor neurons at 18 months of age (B. Malik and L. Greensmith, personal written communication, July 30, 2018, and references 12 and 17). NfL measurements performed on the same platform as for the human samples did not show a significant increase in AR100 mice at 18 months vs littermate controls (n = 9; *p* = 0.45) (figure 1C).

NfL levels are not increased in blood-derived biofluids from 2 independent SBMA cohorts and a well-established mouse model of disease.

### Muscle damage and muscle mass markers are significantly altered in SBMA

**Figure 2:**
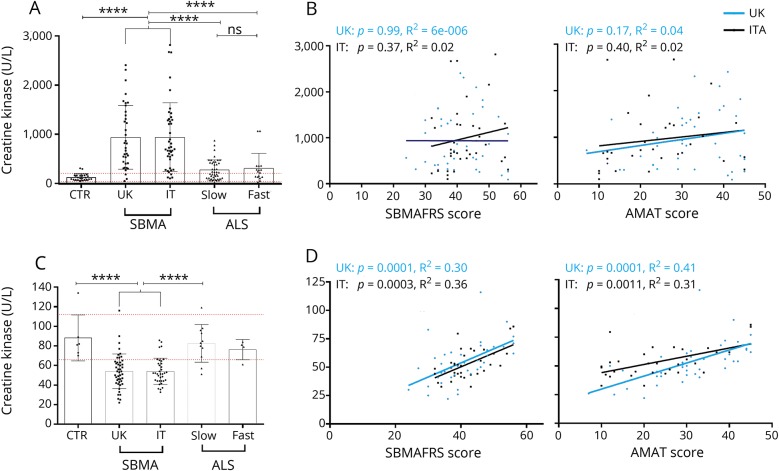
Creatine kinase (CK) and creatinine levels are altered in spinal and bulbar muscular atrophy (SBMA) and correlate with clinical severity (A) CK SBMA plasma concentrations from both UK and Italian cohorts are significantly increased compared to controls (CTR) (*p* < 0.0001) and slow (*p* < 0.0001) and fast (*p* = 0.0003, United Kingdom; *p* = 0.0002, Italy) progressive amyotrophic lateral sclerosis (ALS). (B) CK concentrations of both UK and Italian cohorts do not significantly correlate with the Spinal and Bulbar Muscular Atrophy Functional Rating Scale (SBMAFRS) (left) and Adult Myopathy Assessment Tool (AMAT) (right) clinical severity scores (for SBMAFRS: United Kingdom, *r* = −0.02, *p* = 0.99; Italian, *r* = 0.25, *p* = 0.15; and for AMAT: United Kingdom, *r* = 0.15, *p* = 0.32; IT, *r* = 0.29, *p* = 0.11). (C) Creatinine plasma concentrations from both UK and Italian cohorts are significantly decreased compared to controls (*p* < 0.0001) and slow progressive ALS (*p* < 0.0001). (D) Creatinine concentrations of both UK and Italian cohorts significantly correlate with the SBMAFRS (left) and AMAT (right) clinical severity scores (for SBMAFRS: United Kingdom, *r* = 0.61, *p* < 0.0001; Italy, *r* = 0.49, *p* = 0.0035; and for AMAT: United Kingdom, *r* = 0.75, *p* < 0.0001; Italy, *r* = 0.50, *p* = 0.0044). Blue (United Kingdom) and black (Italy) lines represent the best-fit line. Red dashed lines represent upper and lower extremities of the normal range. Columns indicate mean, error bars indicate SD; ****p < 0.0001; ns *p* > 0.05.

We investigated the levels of markers of muscle damage (CK) and muscle mass (creatinine) in our patient cohorts using standard clinical diagnostic assays. CK levels showed remarkably similar values in both the UK and Italian cohorts (mean 951 and 940 U/L, respectively), and were significantly increased compared to both healthy controls (mean 122 U/L, *p* < 0.0001 for both UK and Italian cohorts) and ALS (*p* < 0.0001 for both UK and Italian cohorts vs slow and fast progressing ALS). Interestingly, CK levels, differently from NfL, did not differ between slow and fast progressing ALS (mean 699 and 701 U/L, respectively, *p* > 0.99) (figure 2A). Creatinine levels were very similar in UK and Italian cohorts and significantly decreased compared to controls and ALS (figure 2C).

### Creatinine levels correlate with SBMA severity

To evaluate disease severity, we used the SBMAFRS, a function rating scale developed for SBMA, and the AMAT score, which detects deficits in functional muscle performance and endurance and has been used as an outcome measure for SBMA clinical trials.^13–15^ While no significant correlation was present between clinical measures and CK (figure 2B), creatinine levels strongly inversely correlated with both SBMAFRS and AMAT in both SBMA cohorts (figure 2D).

## Discussion

We show that NfL concentration in blood is unchanged in patients with SBMA compared with controls, and concentrations do not change over time and are not related to disease severity. The result was confirmed in a well-established mouse model of SBMA. This is surprising given that NfL has been described to be increased in numerous neurodegenerative conditions and mouse models, and cannot simply be imputed solely to the slowly progressive nature of SBMA, as a recent study using the same technique has detected an NfL increase in Charcot-Marie-Tooth disease, another very slowly progressive neuromuscular condition.^10,11,16,18,19^

Conversely, markers of muscle damage (CK) and muscle mass (creatinine) are aberrant in SBMA. CK is elevated compared to both controls and ALS cases, and does not correlate to clinical measures, possibly due to its reflection of more acute muscle damage, whereas creatinine is reduced and significantly correlates with disease severity. A decrease in creatinine has also been shown in a Japanese SBMA cohort to correlate with grip strength and 6-minute walk distance and has also been shown to be lower in patients with SBMA than in patients with ALS who have comparable wasting of muscle mass.^20,21^

Traditionally SBMA has been considered a lower motor neuron (LMN) disease. Indeed, denervation is a crucial neurophysiology finding, and loss of LMN in postmortem tissue has been well-documented.^2,22^ Muscle abnormalities and CK levels elevated beyond those found in ALS have been described in early case series of this disease.^23^ Nonetheless, only recently, analysis of SBMA muscle biopsies, and functional work specifically exploring the role of muscle in mouse models, have led to acknowledging a primary muscle component to be relevant for disease progression and pathogenesis alongside the neurogenic component,^3–9^ and our findings support this.

Finally, SBMA and ALS are in differential diagnosis, and a substantial proportion of patients with SBMA initially receive a diagnosis of ALS, causing distress.^24^ Although genetic clinical testing is the gold standard for SBMA diagnosis, the finding of normal levels of NfL in SBMA could be used in a diagnostic panel of biochemical markers to help differentiate patients presenting with motor neuron deficits.

NfL concentrations in blood were unchanged in 2 cohorts of patients with SBMA and in a mouse model of disease, while markers of muscle damage and mass were altered, the latter showing correlation with clinical measures of disease, suggesting that biomarkers of muscle damage and mass, rather than neuronal damage, should be used to monitor disease progression and outcome. This is consistent with previous observations that a primary myopathic component plays a primary role in the disorder^3–9^ and supports the development of novel disease-modifying agents towards the muscle target and the incorporation of muscle biomarkers to objectively assess outcomes in therapeutic trials in SBMA.

## Glossary

ALS: amyotrophic lateral sclerosis
ALSFRS: Amyotrophic Lateral Sclerosis Functional Rating Scale
AMAT: Adult Myopathy Assessment Tool
AR: androgen receptor
CK: creatine kinase
KD: Kennedy disease
LMN: lower motor neuron
NfL: neurofilament light chain
PRL: progression rate to last visit
SBG: streptavidin-β-galactosidase
SBMA: spinal and bulbar muscular atrophy
SBMAFRS: Spinal and Bulbar Muscular Atrophy Functional Rating Scale
